# Extradigital Glomus Tumor with Atypical Neuritis Presentation

**DOI:** 10.7759/cureus.2794

**Published:** 2018-06-13

**Authors:** Christopher Odom, Brooks Ficke, Nicholas Dahlgren, Harshadkumar A Patel, Katherine Buddemeyer, Chason Farnell, Ashish Shah, Nileshkumar Chaudhari

**Affiliations:** 1 Orthopaedic Surgery, University of Alabama at Birmimgham, Birmingham, USA; 2 Orthopaedic Surgery, Resurgens Orthopaedics, Atlanta, USA; 3 School of Medicine, University of Alabama at Birmingham, Birmingham, USA; 4 Orthopaedic Surgery, University of Alabama at Birmingham, Birmingham, USA

**Keywords:** glomus tumor, wrist, mass, glomus, case report, extradigital

## Abstract

Glomus tumors are rare tumors of the arteriovenous junction that play a role in temperature regulation. They are most commonly found in the subungual finger. We present the case of a 77-year-old female with a chief complaint of a painful mass in her ulnar wrist. The differential diagnosis at the time was broad. Following a detailed history and physical exam, the etiology was believed to be that of a peripheral nerve sheath tumor. The patient was taken to the operating room for resection and biopsy of the mass. Histological evaluation confirmed that the mass was a glomus tumor. Our patient’s symptoms had completely resolved and functional status had improved to baseline by the time of her two-week postoperative clinic visit. This case report demonstrates the many complexities in the diagnosis of a glomus tumor and the important role of surgical treatment in obtaining relief from extradigital glomus tumors.

## Introduction

The glomus body is a contractile neuromyoarterial structure present in the reticular dermis at the arteriovenous junction whose main function is to control body temperature [1]. Glomus tumors are benign tumors arising from a glomus body, and they are commonly found in the subungual finger [2]. The classic clinical presentation consists of severe pain, localized tenderness, and significant cold sensitivity [3]. Given the rare occurrence and broad range of symptoms, making the clinical diagnosis of a glomus tumor outside of its typical subungual location can be difficult. Imaging modalities may not be useful due to the soft-tissue consistency and typical small size of the tumors. We present a report of a patient with a glomus tumor that was discovered in an unusual location. While our suspected diagnosis was incorrect, the proper evaluation and time-sensitive treatment allowed our patient to have a complete resolution of her symptoms.

## Case presentation

A 77-year-old female presented to the orthopedic hand clinic with a three-year history of an extremely sensitive small mass on her right wrist. The mass had subjectively grown over this period of time. The pain had progressively worsened over time, and she had developed significant hypersensitivity to light contact. There was no complaint of cold sensitivity to the mass. The pain occasionally radiated down the ulnar aspect of her wrist. She had no known history of previous trauma to this area; however, she did have a history of squamous cell carcinoma to the dorsal-radial aspect of that hand. This had been treated previously for which she subsequently developed a reflex sympathetic dystrophy (RSD), resulting in a delayed recovery in the range of motion. A previous stellate ganglion block did not provide relief for her RSD, and her range of motion had been slowly progressing with home exercises.

On physical exam, a small round nodule approximately 5 mm x 5 mm was palpable dorsal to the extensor carpi ulnaris and 1 cm proximal to the ulnar styloid. There was significant point tenderness that did not radiate or display a Tinel’s sign. Her imaging included plain films of the affected extremity that showed no abnormality outside of diffuse osteopenia.

**Figure 1:**
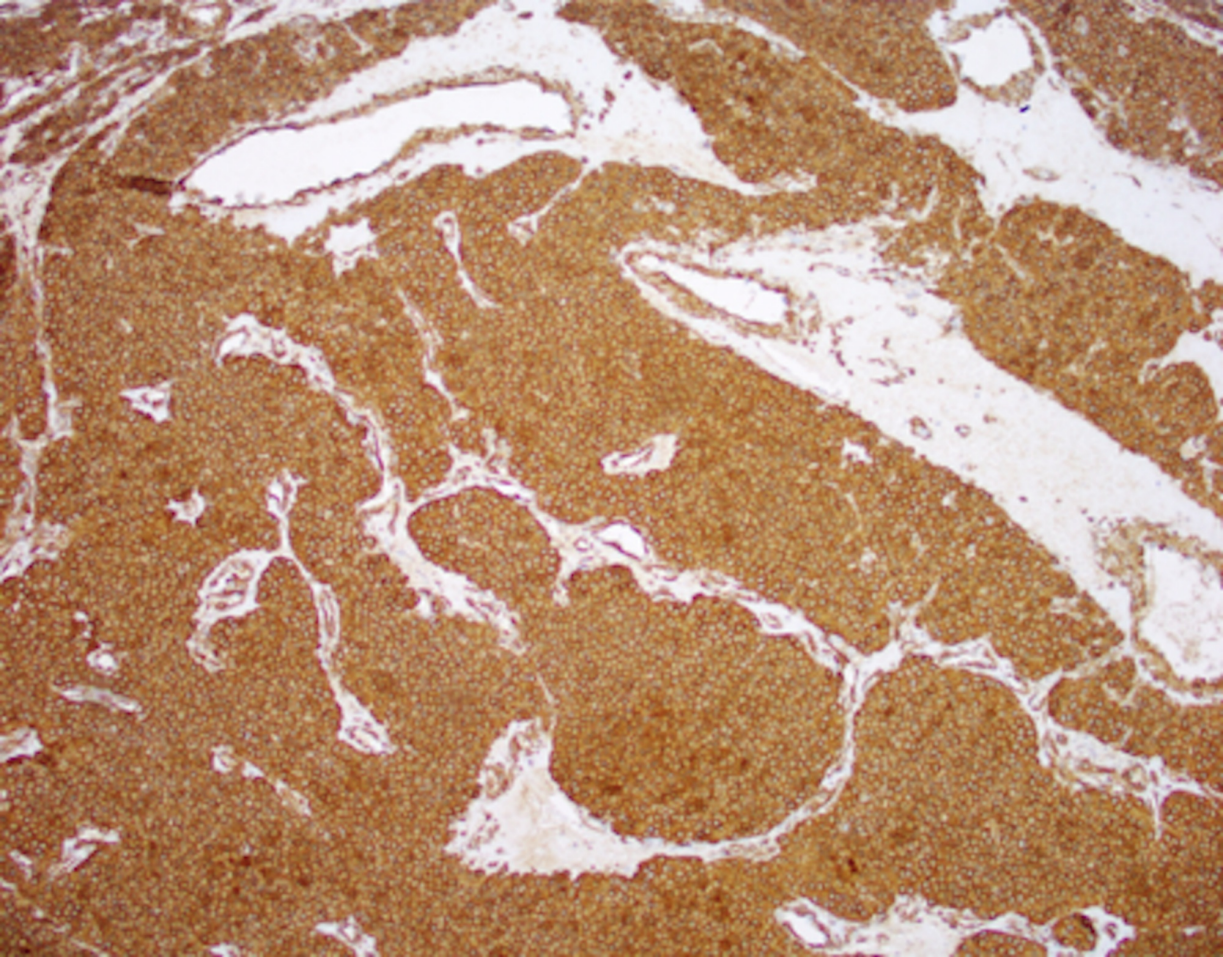
Positive smooth muscle actin at 100x

**Figure 2:**
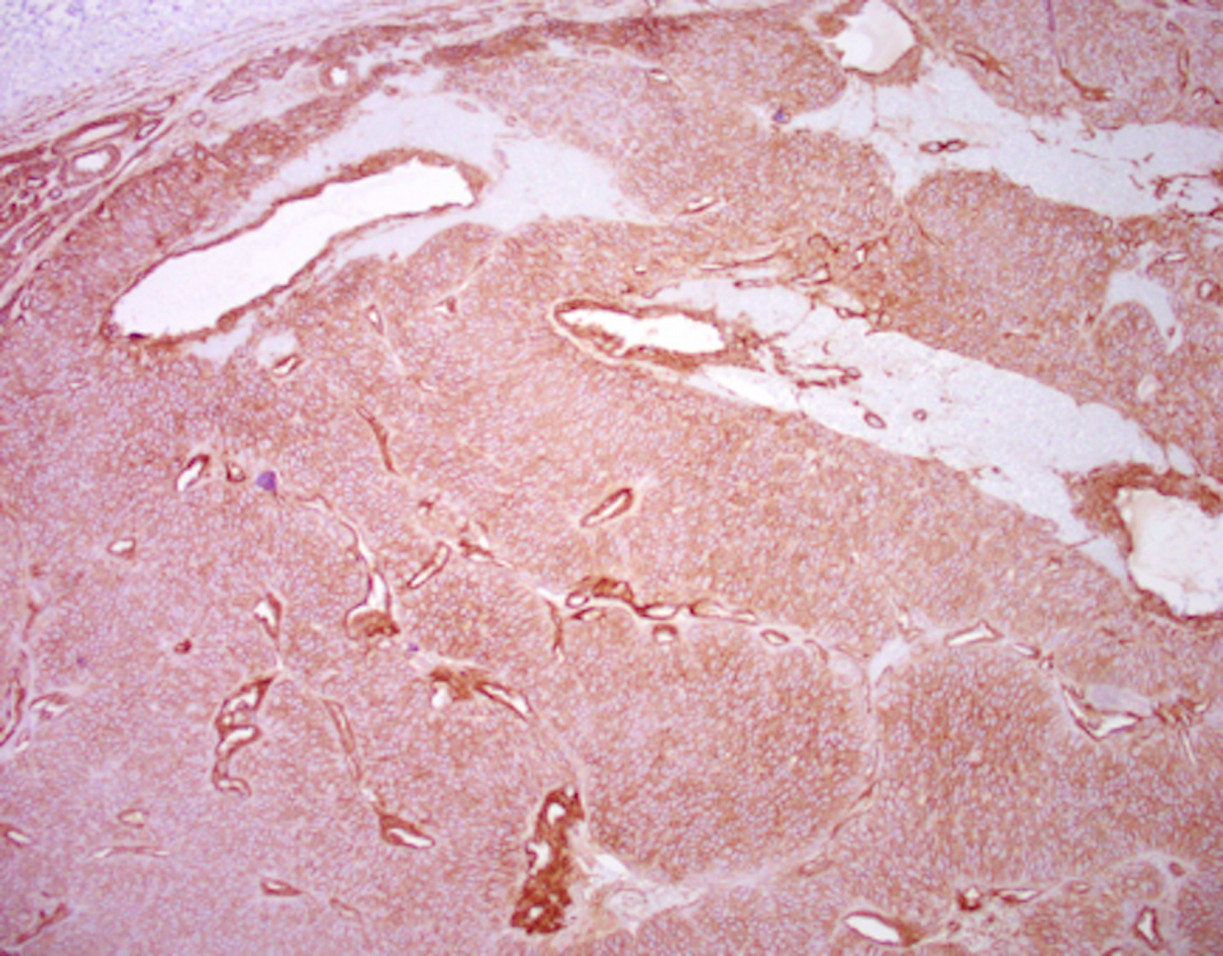
Positive stain for Collagen IV at 100x

The location and exam were consistent with a neuroma that had evolved from a cutaneous nerve or possibly from the dorsal sensory branch of the ulnar nerve. The patient was taken to the operative theatre and deep dissection revealed a maroon-colored mass approximately 5 mm x 5 mm, connected to a cutaneous nerve branch. The nerve and mass were excised and sent for histopathological review. The ulnar nerve and dorsal sensory branch were visualized and confirmed to have no involvement with the mass. At her first postoperative visit, she reported no pain and that she was very satisfied with the results of her surgery. Diagnostic pathological stains were consistent with a glomus tumor. This was confirmed with strong reactivity to immunostaining of type IV collagen and smooth muscle actin (Figures 1, 2).

## Discussion

The three most frequent types of benign mesenchymal tumors in the hand are synovial ganglion cysts (52%), a giant cell tumor of the tendon sheath (11%), and epidermoid cysts (6%) [4]. Though we see these conditions most commonly in the clinic, it remains important to acknowledge the wide variety in the differential diagnosis of a mass around the hand. The majority of glomus tumors present with the classic features of pain with cold sensitivity at the tip of the finger. Prior studies have documented up to 75% to 90% subungual locations for these tumors due to the high concentration of glomus bodies in the area [5]. While an extradigital manifestation is rare, similar glomus tumors in the wrist have been described in previous literature [6-12]. In our case, the location, a previous history of treated squamous cell carcinoma in the symptomatic hand complicated by RSD, the chronicity of symptoms, and non-diagnostic imaging made her diagnosis difficult. It was not until the pathology report that we had a confirmed diagnosis of a glomus tumor.  

Physical exam was significant for point tenderness, although this is extremely non-specific, especially given the location proximal to the wrist joint. A glomus tumor usually is a benign condition that responds favorably to a complete excision, leading to a cure with a low incidence of recurrence [13]. Our patient experienced a resolution of her symptoms immediately following tumor removal. 

**Figure 3:**
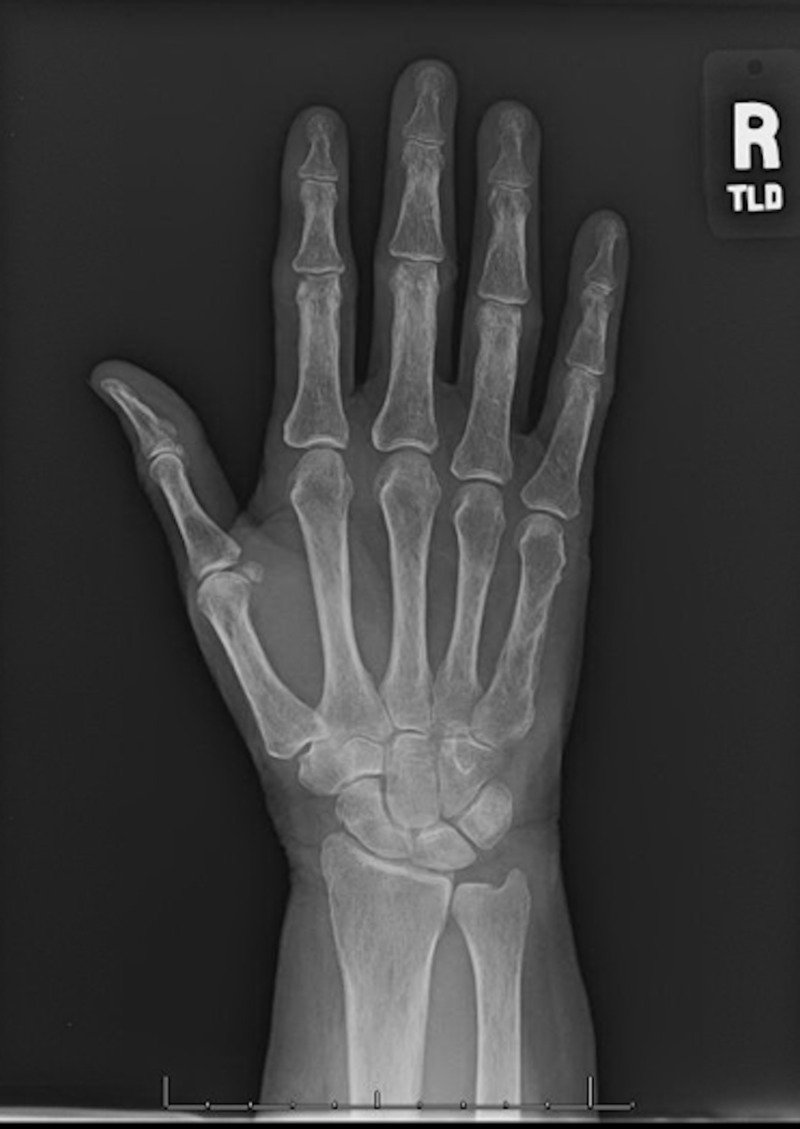
Anteroposterior view of the right hand

**Figure 4:**
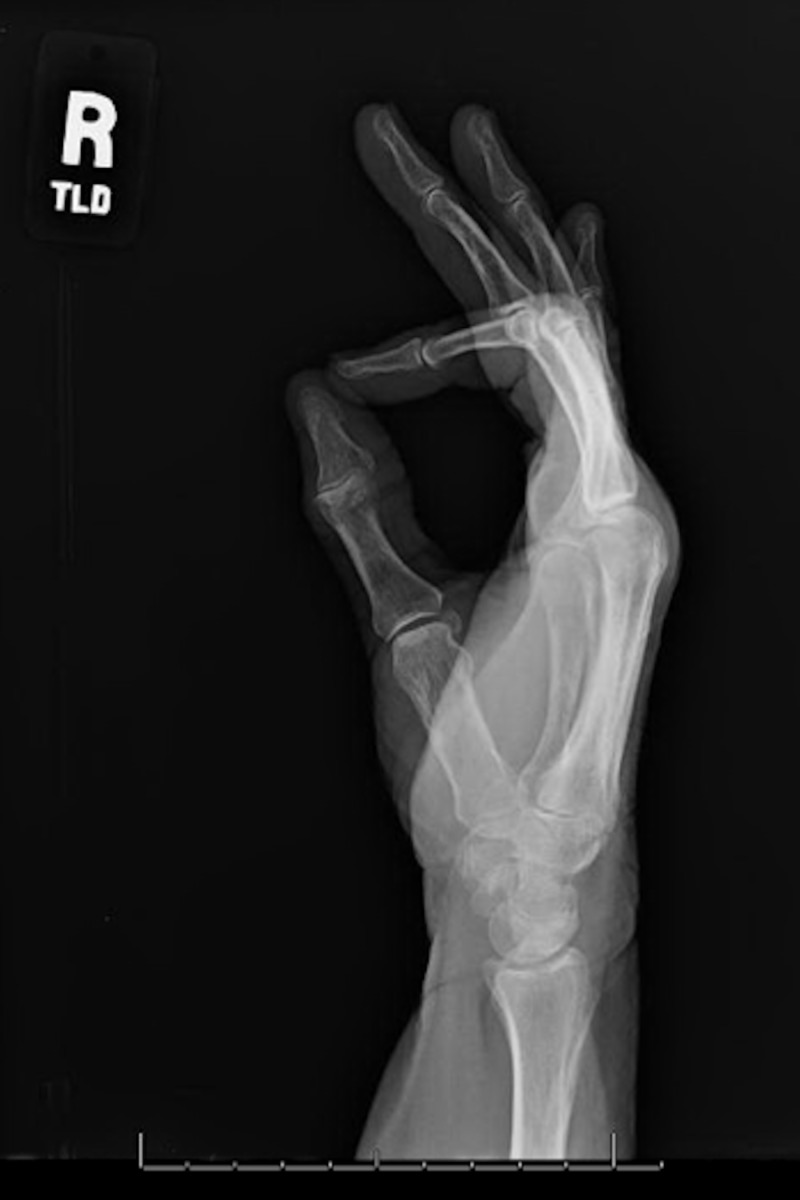
Lateral view of the right hand

Radiographs were obtained to rule out an acute osseous etiology. In subungual glomus tumors, radiographs will occasionally demonstrate scalloping of the terminal phalanx at the site of the lesion [4]. Our patient’s radiographs did not have any significant findings, which was not surprising, given the palpable soft tissue consistency of the mass (Figures 3, 4).

While previous literature has described obtaining magnetic resonance imaging (MRI) as crucial in clinching a diagnosis of glomus tumor early [13], we elected not to obtain the study. A high-quality MRI should define a 5 mm x 5 mm mass, but it would have delayed her definitive treatment and prolonged her painful symptoms. For this reason, we decided to proceed to the operative theatre for excisional biopsy. The results from a cohort of 42 patients with a clinical diagnosis of glomus tumor concluded that the cost of preoperative MRIs with a specificity of only 50% and a negative predictive value of 20% no longer justified the study [14]. Other imaging studies, such as ultrasonography and angiography, have not been shown to produce a specific image for a glomus tumor, often used only for documenting the location and size of the mass [15].

**Figure 5:**
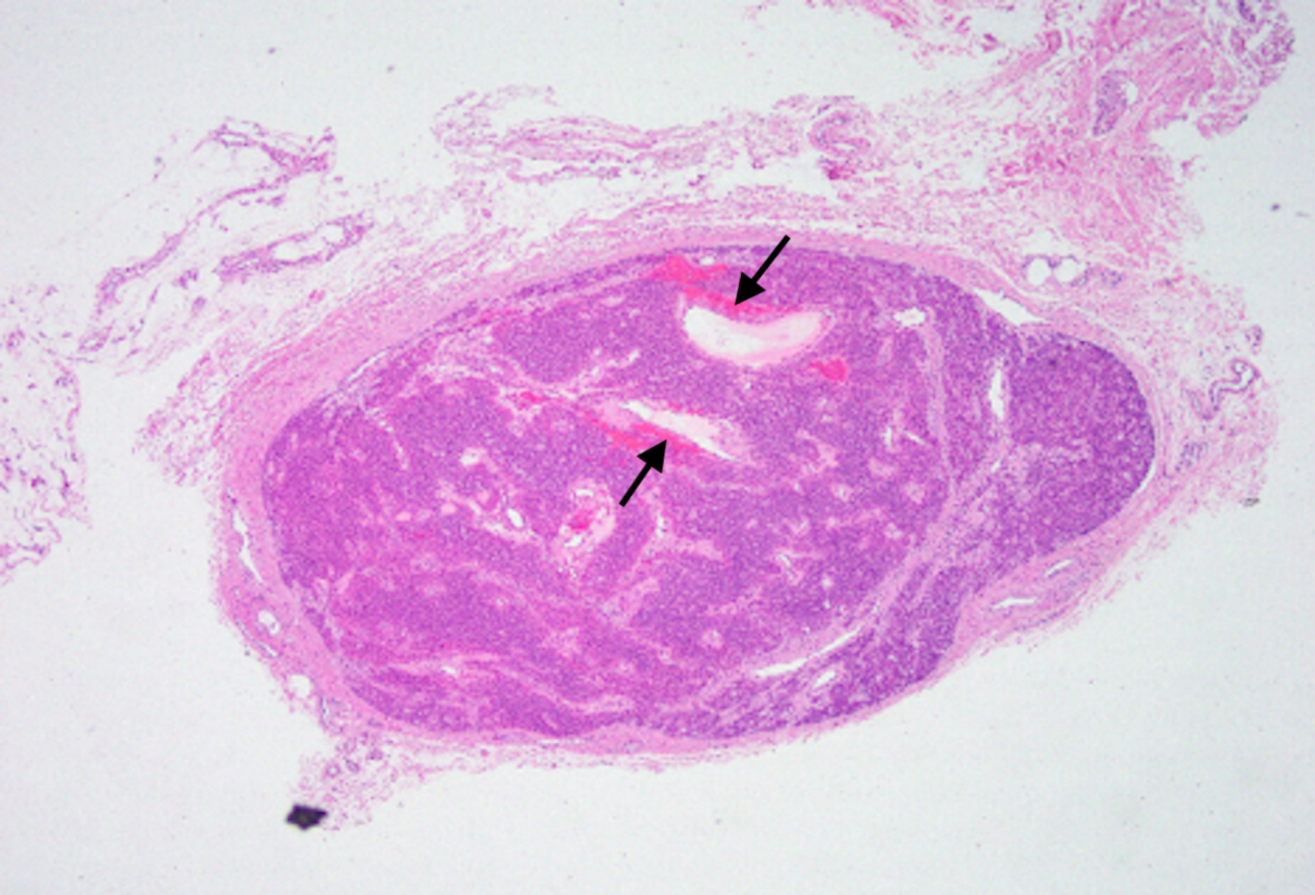
40x magnification of histopathologic sample

The diagnosis of a glomus tumor is confirmed when histology demonstrates cells with a positive expression for CD34 and smooth muscle actin [16]. Three different histologic variants have been reported, including (1) solid, with poor vasculature and scant smooth muscle component, (2) angiomatoid (glomangioma) with a predominant vascular component, and (3) glomangiomyoma with prominent vascular and smooth muscle components [17]. As seen in Figure 5, there was a prominent vascular component to our patient’s tumor.

There is a broad differential for soft tissue masses found throughout the hand and upper extremity. This case is an example of a unique discovery from an unexpected etiology. Our scenario also demonstrates how we, as both clinicians and surgeons, have many tools at our disposal; by avoiding unnecessary studies, we can provide our patients with the efficient, diagnostic, and hopefully pain-relieving service that they desire.

## Conclusions

A diagnosis of an extradigital glomus tumor is difficult to obtain in the clinical setting. The above case illustrates many of the features that make the diagnosis of an extradigital glomus tumor challenging. It is important to have the suspicion of a glomus tumor in the clinician’s differential because expedited and relatively simple treatment can provide quick relief to a significant problem.
